# Supplementary material for: Hybrid Models and Biological Model Reduction with PyDSTool
Source: PLoS Comput Biol. 2012 Aug 9;8(8):e1002628. doi: 10.1371/journal.pcbi.1002628 (PMC3415397; doi:10.1371/journal.pcbi.1002628)
Supplement: Text S4 — Complete source code for the PyDSTool package (version 0.88.120504). Includes API documentation and help files linking to web pages. This file is identical to the current public release on Sourceforge.net. (ZIP) [file pcbi.1002628.s004.zip › PyDSTool/html/PyDSTool.Toolbox.optimizers.criterion.composite_criteria.AndComposition-class.html]

xml version="1.0" encoding="ascii"?


PyDSTool.Toolbox.optimizers.criterion.composite\_criteria.AndComposition


| Home | Trees | Indices | Help | PyDSTool |
| --- | --- | --- | --- | --- |

Package PyDSTool ::
Package Toolbox ::
Package optimizers ::
Package criterion ::
Module composite\_criteria ::
Class AndComposition

[hide private]

[frames] | no frames]

# Class AndComposition

source code

```
object --+
         |
        AndComposition
```

---

Compose several criteria with an and rule


|  |  |
| --- | --- |
| Instance Methods | [hide private] |

|  |  |
| --- | --- |
| \_\_init\_\_(self, \*args, \*\*kwargs)  Collects the different criteria | source code |

|  |  |
| --- | --- |
| \_\_call\_\_(self, state, \*\*kwargs)  Evaluates each criterion (no lazy evaluation) and returns True if one of them is True | source code |

**Inherited from `object`**:
`__delattr__`,
`__getattribute__`,
`__hash__`,
`__new__`,
`__reduce__`,
`__reduce_ex__`,
`__repr__`,
`__setattr__`,
`__str__`


|  |  |
| --- | --- |
| Properties | [hide private] |

**Inherited from `object`**:
`__class__`


|  |  |
| --- | --- |
| Method Details | [hide private] |

|  |  |
| --- | --- |
| \_\_init\_\_(self, \*args, \*\*kwargs)  *(Constructor)* | source code |

Collects the different criteria

Overrides: object.\_\_init\_\_

  


| Home | Trees | Indices | Help | PyDSTool |
| --- | --- | --- | --- | --- |

|  |  |
| --- | --- |
| Generated by Epydoc 3.0.1 on Fri May 4 15:24:09 2012 | http://epydoc.sourceforge.net |
